# Supplementary material for: The FLAME-accelerated signalling tool (FaST) for facile parallelisation of flexible agent-based models of cell signalling
Source: NPJ Syst Biol Appl. 2020 Apr 20;6:10. doi: 10.1038/s41540-020-0128-x (PMC7170865; doi:10.1038/s41540-020-0128-x)
Supplement: Supplementary file 1 — Supplementary Material [file 41540_2020_128_MOESM1_ESM.pdf]

Supplementary information for:
The FLAME-accelerated Signalling Tool (FaST) for facile parallelisation of flexible agent-based models of cell signalling

Gavin Fullstone*,[1,2], Cristiano Guttà[1], Amatus Beyer[1] and Markus Rehm*,[1,2]

Table of Contents

Supplementary Note 1. FLAME: An approach to parallelisation of agent-based applications .. 1

Agent-based modelling with FLAME 1

Supplementary Figure 1. Agent-based modelling of soluble-soluble reactions is able to reproduce mass action kinetics. 2

Supplementary Figure 2. Agent-based modelling of membrane-soluble reactions is able to reproduce mass action kinetics. 3

Supplementary Figure 3. Agent-based modelling of membrane-membrane reactions is able to reproduce mass action kinetics. 4

Supplementary Note 2. Reproduction of an established ODE model using ABM 5

Supplementary Table 1. List of Agents and Concentrations 5

Supplementary Table 2. List of Reactions and Rates 6

Supplementary Note 3. Choosing an appropriate time step for FaST 6

Supplementary Note 1. FLAME: An approach to parallelisation of agent-based applications

In order to improve the scaling up of agent-based modelling, a number of different computational approaches are available. The scale of an agent-based simulation run on a CPU is limited by the amount of memory (primarily limits the number of agents) and computational power available (limits the number of agents and the functions that each agent can perform). In order to increase simulation size, distributed or parallel computing can be used, where memory and computation is spread across multiple processing units. This can be done with CPUs or alternatively with GPUs, such as those in computer graphics cards. GPUs usually offer greater computational power than CPUs and therefore are an attractive alternative for large-scale data processing, however they are limited by the fixed amount of memory. In agent-based modelling, this makes them very attractive for smaller, less memory intensive simulations but may limit their use in larger simulations or memory-intensive simulations such as ABM-ODE hybrids. Parallel computing with CPUs removes the theoretical upper limit of simulation size, based on memory and computational demands. CPU parallelisation uses the Message Passing Interface (MPI), which allows CPUs to send data to and receive data from other CPUs. However, whilst reading and writing locally stored data is relatively fast, messages passed through the MPI are comparatively slow and limited by the speed of the interconnect between CPUs. Therefore, careful optimisation of parallel codes is required to obtain maximum speed up of the simulation.

Agent-based modelling with FLAME

The major drawback of GPU and parallel CPU agent-based simulation is that it normally requires extensive knowledge of CUDA or MPI coding respectively and careful optimisation.

FLAME and FLAME GPU use a communicating X-machine approach to agent-based modelling. The user defines agents; their respective memory variables; their respective functions to carry out and input/output requirements for those functions in the form of messages. This is achieved in a basic form using XMML (X-Machine Markup Language). The functions themselves are then coded in a separate function file or files coded in the C language. FLAME and the FLAME GPU Software Development Kit (SDK) utilises these two user-generated codes to construct iterative-based executable models in either for CPUs or GPUs respectively. FLAME and FLAME GPU use messages for communications between agents. The XMML file declares these messages and the variables stored in them. The CPU version of FLAME stores these messages on message boards. Functions that require data from other agents read through all the messages on a particular message board and by using filtering can access the required data efficiently. In serial, agents can read and write to this board rapidly as it is maintained in the local memory. However, in parallel each core has its own message board that must be synced with the other boards. When a function adds data to a message board, a message is generated to update the other message boards. Whilst messages are in transit through the message-passing interface, no function can read data from the message board. FLAME's approach to parallelisation uses a scheduler that prioritises work that will generate communications between CPUs. Whilst the messages are sent in the background, it will then perform all possible work not dependent on those communications, in order to reduce overhead times associated with parallelisation. This method of parallelisation optimisation has the additional advantage that the addition or removal of functions does not require re-optimisation and therefore allows a *plug and play* approach to adding functionality.

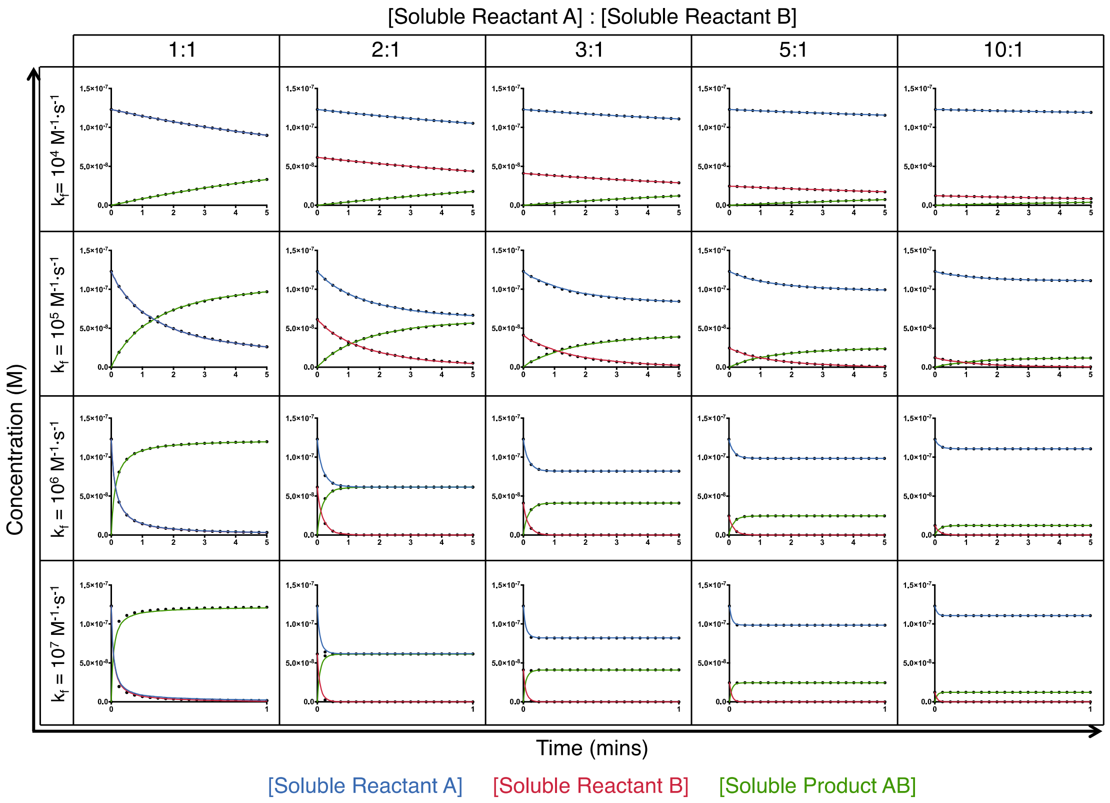

Supplementary Figure 1. Agent-based modelling of soluble-soluble reactions is able to reproduce mass action kinetics. The reaction of two soluble molecules were simulated with ABM and the change in reactant and product concentration was observed (solid colour lines) and compared to equivalent ODE models (dashed black lines) for k_f values of 10^4 $M^{-1}·s^{-1}$, 10^5 $M^{-1}·s^{-1}$, 10^6 $M^{-1}·s^{-1}$ and 10^7 $M^{-1}·s^{-1}$ and different concentration ratios of A to B ([A]:[B]). All simulations were for 5 minutes, the time step Δt for particle diffusion in all simulations was

0.0001 s and for reactions was 0.05 s. The diffusion coefficients used were 30 μm^2·s^{-1}. Each point represents mean from three independent simulations, consequent error bars are too small to be plotted.

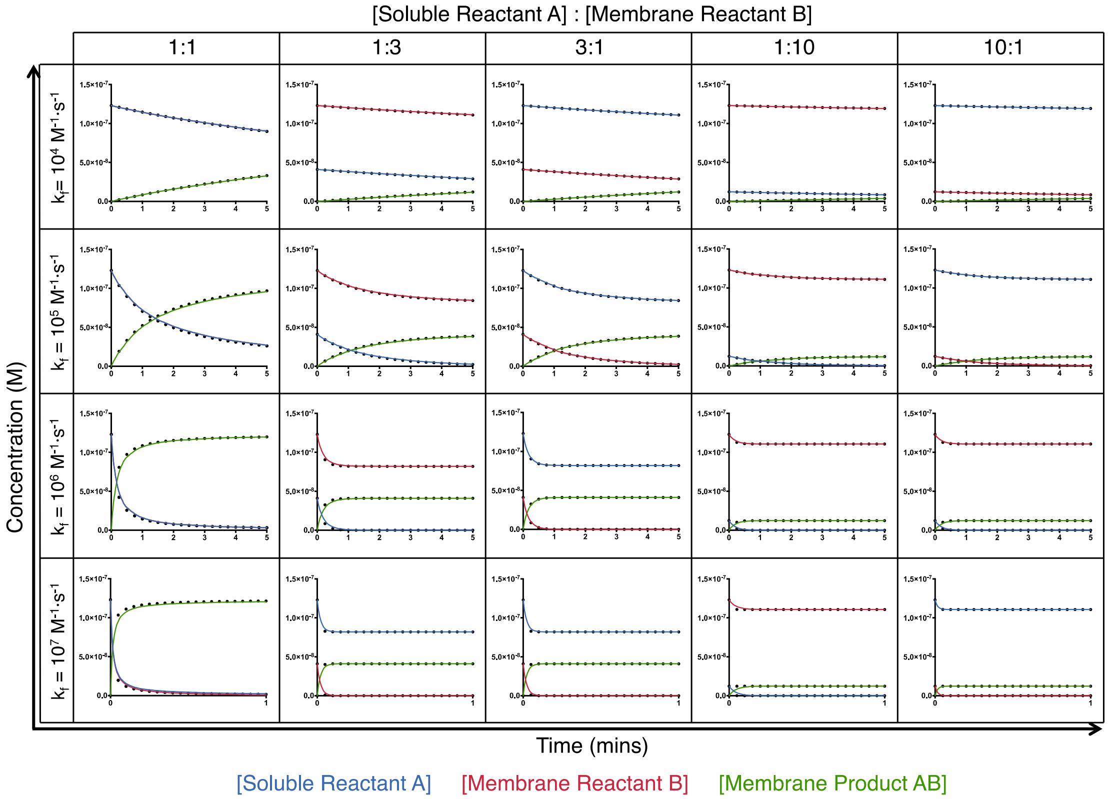

Supplementary Figure 2. Agent-based modelling of membrane-soluble reactions is able to reproduce mass action kinetics. The reaction of a soluble and a membrane-bound molecule were simulated with ABM and the change in reactant and product concentration was observed (solid colour lines) and compared to equivalent ODE models (dashed black lines) for k_f values of 10^4 M^{-1}·s^{-1}, 10^5 M^{-1}·s^{-1}, 10^6 M^{-1}·s^{-1} and 10^7 M^{-1}·s^{-1} and different concentration ratios of A to B ([A]:[B]). All simulations were for 5 minutes, the time step Δt for particle diffusion in all simulations was 0.0001 s and for reactions was 0.05 s. The diffusion coefficients used were 30 μm^2·s^{-1} for the soluble A and 0.3 μm^2·s^{-1} for the membrane-bound B. Each point represents the mean from three independent simulations, consequent error bars are too small to be plotted.

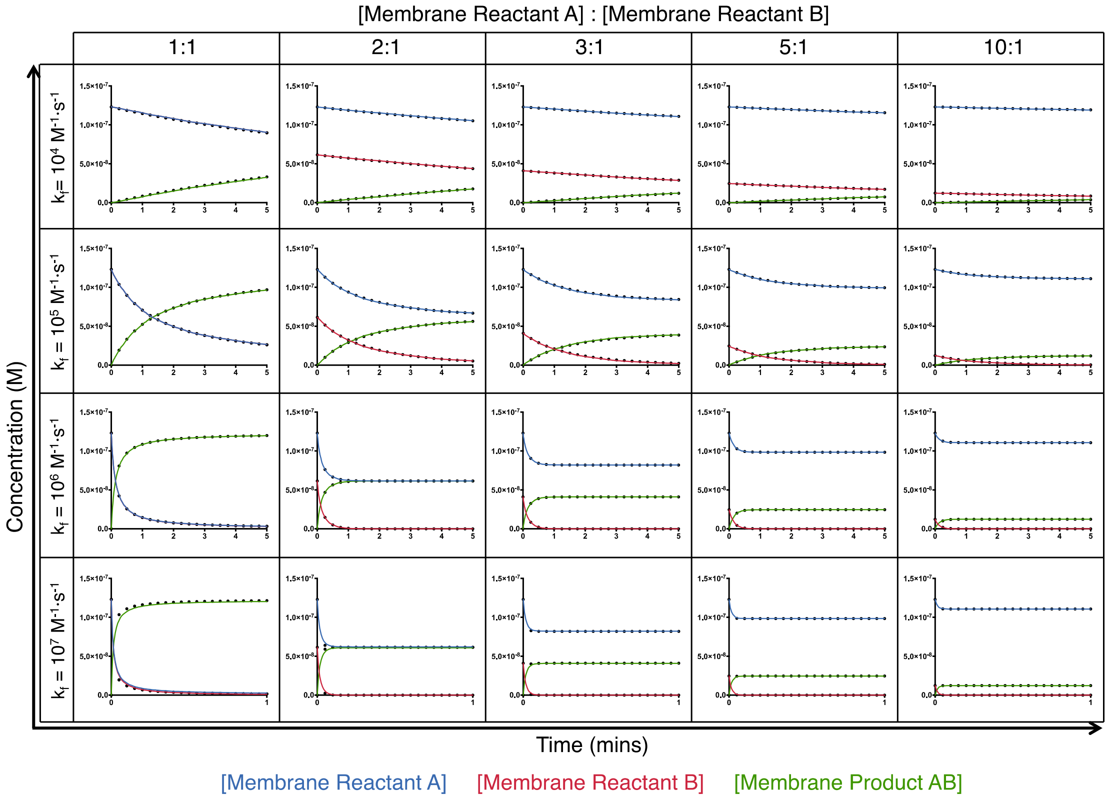

Supplementary Figure 3. Agent-based modelling of membrane-membrane reactions is able to reproduce mass action kinetics. The reaction of two membrane-bound molecules were simulated with ABM and the change in reactant and product concentration was observed (solid colour lines) and compared to equivalent ODE models (dashed black lines) for k_f values of 10^4 M^{-1}·s^{-1}, 10^5 M^{-1}·s^{-1}, 10^6 M^{-1}·s^{-1} and 10^7 M^{-1}·s^{-1} and different concentration ratios of A to B ([A]:[B]). All simulations were for 5 minutes, the time step Δt for particle diffusion in all simulations was 0.0001 s and for reactions was 0.05 s. The diffusion coefficients used were 0.3 μm^2·s^{-1}. Each point represents mean from three independent simulations, consequent error bars are too small to be plotted.

Supplementary Note 2. Reproduction of an established ODE model using ABM

The ODE model of apoptosis execution is a simplified representation of this pathway, where pro-caspase 9 is activated according to kinetic data representing an entire process involving cytochrome c release, resultant formation of a platform called the apoptosome and subsequent apoptosome-dependent pro-caspase 9 activation to caspase 9. We adapted this original model with two minor alterations to create the modified ODE and ABM models. First, SMAC was always treated as a dimer in its native form; therefore its concentration and the reactions were adjusted accordingly. Second, the cleavage of XIAP by caspase 3 was removed from the existing model. The resultant reaction schematic modelled in this paper is included in Figure 3a. We took the original concentrations and reaction kinetic data from the work of Rehm and colleagues, as summarised in Supplementary Tables 1-2, to construct the agents.txt and reactions.txt files required by the FLAME signalling tool. These files are also available with FaST.

Supplementary Table 1. List of Agents and Concentrations

ID	Name	Concentration (M)	Diffusion Coefficient ($m^2 \cdot s^{-1}$)	Description
1	PC9	3×10^{-8}	2.024×10^{-11}	Pro-Caspase 9
2	C9	0	2.024×10^{-11}	Caspase 9
3	C9P	0	2.462×10^{-11}	Processed Caspase 9
4	PC3	1.2×10^{-7}	2.201×10^{-11}	Pro-Caspase 3
5	C3	0	1.860×10^{-11}	Caspase 3
6	XIAP	6.3×10^{-8}	1.893×10^{-11}	XIAP
7	MitoSMAC	6.3×10^{-8}	2.462×10^{-11}	Mitochondrial SMAC
8	SMAC	0	2.461×10^{-11}	Cytoplasmic SMAC
9	XIAP-C3	0	1.490×10^{-11}	XIAP-Caspase 3
10	XIAP-C9	0	1.551×10^{-11}	XIAP-Caspase 9
11	XIAP-C3-C9	0	1.331×10^{-11}	XIAP-Caspase 3-Caspase 9
12	XIAP-SMAC	0	1.671×10^{-11}	XIAP-SMAC
13	Substrate	1.00×10^{-6}	1.905×10^{-11}	C3 Substrate
14	cSubstrate	0	2.400×10^{-11}	C3-Cleaved Substrate

All concentrations were taken from the publication of Rehm et al., 2006. SMAC was here considered constitutively a dimer and so the concentration was halved accordingly[1]. Diffusion coefficients were calculated relative to GFP (27 kDa; 2.4×10^{-11} $m^2 \cdot s^{-1}$) according to the methods described.

Supplementary Table 2. List of Reactions and Rates

#	Reaction	Forward Rate	Reverse Rate
1	PC9 → C9	5.022×10^{-3} s^{-1}	
2	MitoSMAC → SMAC	1.65×10^{-3} s^{-1}	
3	C9 + PC3 → C9 + C3	1×10^{5} M^{-1}·s^{-1}	
4	C9 + C3 → C9P + C3	2×10^{5} M^{-1}·s^{-1}	
5	C9P + PC3 → C9P + C3	8×10^{5} M^{-1}·s^{-1}	
6	C3 + PC3 → C3 + C3	4×10^{4} M^{-1}·s^{-1}	
7	C9 + XIAP ↔ XIAPC9	2.6×10^{6} M^{-1}·s^{-1}	2.4×10^{-3} s^{-1}
8	C3 + XIAP ↔ XIAPC3	2.6×10^{6} M^{-1}·s^{-1}	2.4×10^{-3} s^{-1}
9	C9 + XIAPC3 ↔ XIAPC3C9	2.6×10^{6} M^{-1}·s^{-1}	2.4×10^{-3} s^{-1}
10	C3 + XIAPC9 ↔ XIAPC3C9	2.6×10^{6} M^{-1}·s^{-1}	2.4×10^{-3} s^{-1}
11	XIAP + SMAC ↔ XIAPSMAC	7×10^{6} M^{-1}·s^{-1}	2.21×10^{-3} s^{-1}
12	XIAPC9 + SMAC → XIAPSMAC + C9	7×10^{6} M^{-1}·s^{-1}	
13	XIAPC3 + SMAC → XIAPSMAC + C3	7×10^{6} M^{-1}·s^{-1}	
14	XIAPC3C9 + SMAC → XIAPSMAC + C3	7×10^{6} M^{-1}·s^{-1}	
15	C3 + Substrate → C3 + cSubstrate	2×10^{5} M^{-1}·s^{-1}	
16	C9 → DEGRADED	9.67×10^{-5} s^{-1}	
17	C3 → DEGRADED	9.67×10^{-5} s^{-1}	
18	C9P → DEGRADED	9.67×10^{-5} s^{-1}	
19	XIAPC9 → DEGRADED	5.78×10^{-4} s^{-1}	
20	XIAPC3 → DEGRADED	5.78×10^{-4} s^{-1}	
21	XIAPC3C9 → DEGRADED	5.78×10^{-4} s^{-1}	
22	XIAPSMAC → DEGRADED	5.78×10^{-4} s^{-1}	
23	SMAC → DEGRADED	9.67×10^{-5} s^{-1}	

All reaction rates were used as in the publication of Rehm *et al.*, 2006, except for the reaction rates 11-14 which were taken from Huang *et al.*, 2003[2]. The reaction rate for equations 1-2 were converted from half times in Rehm *et al.*, 2006 by the relation $k = 0.693/t_{1/2}$ where $t_{1/2}$ is the half time[1].

Supplementary Note 3. Choosing an appropriate time step for FaST

FaST uses discrete time steps for Brownian motion and reactions, with reactions being driven by the relative proximity of two molecules after diffusion. The choice of time step is a critical decision in maintaining the balance between efficient runtimes whilst retaining accuracy. Generally, the algorithms in FaST will be more accurate with a shorter time step, but will also be slower. The choice of time step should be made dependent on the modelled system, considering the diffusion length, geometry, reaction rates and concentration of reactants. Generally, the maximum diffusion length (calculated as 3σ, from Equation 2) a molecule should take in a single iteration should be an order of magnitude below the characteristic length of the geometry. In FaST simulations, an option is included to maintain different time steps for Brownian motion and reactions. Brownian motion has a generally much lower computational burden than reactions and so this enables the user to accurately model particle motion whilst retaining computationally efficient reaction time steps. For reactions, the binding radius and unbinding radius is calculated relative to the time step (Equations 7-9, 11 and 15). Similar to the diffusion length, the binding and unbinding radii should be significantly smaller than the characteristic length of the geometry. Moreover, the assumptions used in FaST require that the probability of multiple potential reactants occurring within the binding radius of any giving molecule is infinitesimally small. In the event of this occurring the molecule will choose to bind to only one of the reactants. This is an acceptable correction when such

events are rare, however when these events are relatively common then FaST will begin to create slower kinetics compared to the original reaction rate used as an input. The likelihood of this happening is directly related to the binding radius and concentration of reactants. The user is advised to trial shorter time steps that fit the above considerations and increase it until unacceptable decreases in accuracy occur.

References

1. Rehm, M., Huber, H. J., Dussmann, H. & Prehn, J. H. M. Systems analysis of effector caspase activation and its control by X-linked inhibitor of apoptosis protein. *EMBO J.* **25**, 4338 LP – 4349 (2006).
2. Huang, Y. H., Rich, R. L., Myszka, D. G. & Wu, H. Requirement of both the second and third BIR domains for the relief of X-linked inhibitor of apoptosis protein (XIAP)-mediated caspase inhibition by Smac. *J. Biol. Chem.* **278**, 49517–49522 (2003).
